# Dissecting the interaction between HSP70 and vascular contraction: role of $\hbox{Ca}^{2+}$ handling mechanisms

**DOI:** 10.1038/s41598-021-80966-6

**Published:** 2021-01-14

**Authors:** Amanda A. de Oliveira, Fernanda Priviero, Rita C. Tostes, R. Clinton Webb, Kenia P. Nunes

**Affiliations:** 1 Laboratory of Vascular Physiology, Department of Biomedical and Chemical Engineering and Sciences, Florida Institute of Technology, Melbourne, USA; 2 Department of Physiology, Augusta University, Augusta, USA; 3 Department of Cell Biology and Anatomy, University of South Carolina, Columbia, USA; 4 Department of Pharmacology, Ribeirao Preto Medical School, University of Sao Paulo, Sao Paulo, Brazil

**Keywords:** Cardiovascular biology, Calcium signalling

## Abstract

Heat-shock protein 70 (HSP70) is a ubiquitously expressed molecular chaperone with various biological functions. Recently, we demonstrated that HSP70 is key for adequate vascular reactivity. However, the specific mechanisms targeted by HSP70 to assist in this process remain elusive. Since there is a wealth of evidence connecting HSP70 to calcium ($\hbox {Ca}^{2+}$), a master regulator of contraction, we designed this study to investigate whether blockade of HSP70 disrupts vascular contraction via impairment of ${\text{Ca}}^{2+}$ handling mechanisms. We performed functional studies in aortas isolated from male Sprague Dawley rats in the presence or absence of exogenous $\hbox {Ca}^{2+}$, and we determined the effects of VER155008, an inhibitor of HSP70, on $\hbox {Ca}^{2+}$ handling as well as key mechanisms that regulate vascular contraction. Changes in the intracellular concentration of $\hbox {Ca}^{2+}$ were measured with a biochemical assay kit. We report that blockade of HSP70 leads to $\hbox {Ca}^{2+}$ mishandling in aorta stimulated with phenylephrine, decreasing both phasic and tonic contractions. Importantly, in $\hbox {Ca}^{2+}$ free Krebs’ solution, inhibition of HSP70 only reduced the $\hbox {E}_{\mathrm{max}}$ of the phasic contraction if the protein was blocked before IP3r-mediated $\hbox {Ca}^{2+}$ release, suggesting that HSP70 has a positive effect towards this receptor. Corroborating this statement, VER155008 did not potentiate an IP3r inhibitor’s outcomes, even with partial blockade. In another set of experiments, the inhibition of HSP70 attenuated the amplitude of the tonic contraction independently of the moment VER155008 was added to the chamber (i.e., whether it was before or after IP3r-mediated phasic contraction). More compelling, following re-addition of $\hbox {Ca}^{2+}$, VER155008 amplified the inhibitory effects of a voltage-dependent $\hbox {Ca}^{2+}$ channel blocker, but not of a voltage-independent $\hbox {Ca}^{2+}$ channel inhibitor, indicating that HSP70 has a positive impact on the latter. Lastly, the mechanism by which HSP70 modulates vascular contraction does not involve the $\hbox {Ca}^{2+}$ sensitizer protein, Rho-kinase, nor the SERCA pump, as blockade of these proteins in the presence of VER155008 almost abolished contraction. In summary, our findings shed light on the processes targeted by HSP70 during vascular contraction and open research avenues for potential new mechanisms in vascular diseases.

## Introduction

The primary function of vascular smooth muscle cells, vasoconstriction, helps to sustain arterial tone, and consequently, impacts blood pressure^1^. While changes in the tonus of small resistant arteries directly affect this process, large conducting arteries, such as the aorta, might contribute to blood pressure regulation by transferring pulsatility into the microcirculation^2,3^. Heat-shock protein 70 (HSP70), a molecular chaperone, is an emerging player in vascular physiology as its pharmacological blockade weakens phenylephrine (PE)-induced contraction in isolated aortas^4^. However, the specific mechanism(s) targeted by HSP70 to positively modulate this process is/are mostly unknown, especially considering that, unlike small HSPs, HSP70 does not affect the actin-myosin complex^5^. Therefore, further investigating the interplay between HSP70 and vascular contraction is of utmost importance as it has the potential to unveil target(s) for the treatment of vascular complications associated with chronic conditions, such as diabetes and hypertension.

The presence of $\hbox {Ca}^{2+}$ is a *sine qua non* condition for contraction in all muscle types^6^, and interestingly, there is a wealth of evidence linking HSP70 to $\hbox {Ca}^{2+}$. In fact, previous studies have shown that the genetic deletion of the inducible HSP70 genes affects $\hbox {Ca}^{2+}$ homeostasis, and worsens cardiac and skeletal muscle function^7,8^. Additionally, not only the ATPase domain of HSP70 binds two $\hbox {Ca}^{2+}$ ions^9^, but also changes in the intracellular concentration of $\hbox {Ca}^{2+}$ induce the expression of HSP70^10,11^. However, it is yet-to-be-determined if HSP70 affects $\hbox {Ca}^{2+}$ dynamics in vascular smooth muscle. The cytosolic levels of free $\hbox {Ca}^{2+}$ are directly linked to the degree of contraction elicited by various agonists^12–14^. In this sense, specific protocols can indirectly assess fluctuations in $\hbox {Ca}^{2+}$ levels. For example, the $\alpha -1$ adrenergic agonist, PE, generates a well-characterized biphasic contraction curve in isolated aortas, reflecting the two-part increase in cytosolic levels of $\hbox {Ca}^{2+}$^12^. Specifically, inositol trisphosphate receptor (IP3r)-mediated $\hbox {Ca}^{2+}$ efflux from the sarcoplasmic reticulum (SR) stimulates the first part of the contraction (fast/phasic) and $\hbox {Ca}^{2+}$ influx, either via voltage-dependent or -independent plasmalemmal $\hbox {Ca}^{2+}$ channels, mediates the second part of contraction (prolonged/tonic)^12,15^. Remarkably, depletion of $\hbox {Ca}^{2+}$ stores from the SR connects this biphasic response as it gates $\hbox {Ca}^{2+}$ influx in a process known as store-operated $\hbox {Ca}^{2+}$ entry^16^. In fact, the protein STIM1 acts as a sensor for the SR $\hbox {Ca}^{2+}$ stores and activates $\hbox {Ca}^{2+}$ release-activated (CRAC) channels, such as Orai1^17,18^. Also, Rho-kinase, a downstream RhoA target, modulates contraction by promoting $\hbox {Ca}^{2+}$ sensitization^19–21^. To add another layer of complexity, the cytosolic levels of free $\hbox {Ca}^{2+}$ also rely on the SR $\hbox {Ca}^{2+}$ ATPase (SERCA) pump, which transfers $\hbox {Ca}^{2+}$ into the SR^22^. Thus, it is clear that, following stimulation with PE, vascular smooth muscle contraction depends on $\hbox {Ca}^{2+}$ release from different compartments.

Based on this previous knowledge, we designed this study to investigate whether blockade of HSP70 impacts vascular contraction by impairing $\hbox {Ca}^{2+}$ handling mechanisms. To achieve such a goal, we performed functional studies in a wire myograph using aorta isolated from male Sprague Dawley rats. Experiments were conducted in the presence or absence of exogenous $\hbox {Ca}^{2+}$, and the effects of VER155008, a pharmacological inhibitor of HSP70, on $\hbox {Ca}^{2+}$ handling mechanisms were determined. Changes in the intracellular concentration of $\hbox {Ca}^{2+}$ were evaluated with a biochemical assay kit. Here, we report that blockade of HSP70 leads to vascular $\hbox {Ca}^{2+}$ mishandling. Specifically, we dissected that inhibition of HSP70 affects (a) phasic vascular contraction via crosstalk with IP3r-mediated intracellular $\hbox {Ca}^{2+}$ release and (b) tonic contraction through a complex interaction with voltage-independent $\hbox {Ca}^{2+}$ channels-facilitated $\hbox {Ca}^{2+}$ influx. Together, our findings shed light on the processes targeted by HSP70 in order to assist in vascular contraction, and open research avenues for potential new mechanisms in cardiovascular and metabolic diseases-associated vascular complications.

## Results

### Impact of HSP70 blockade in the total concentration of $\hbox {Ca}^{2+}$ in PE-stimulated aorta

**Figure 1:**
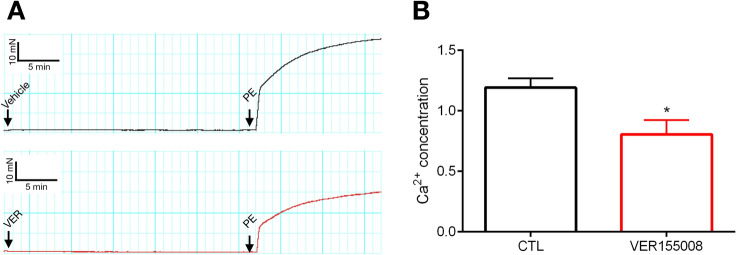
HSP70 contributes to $\hbox {Ca}^{2+}$ handling in phenylephrine-stimulated aorta. (**A**) Representative force tracing (time vs. force) for samples incubated with vehicle (CTL) or VER1550008 ($10\,\upmu \hbox {mol/l}$) for 30 min and challenged with phenylephrine ($10\,\upmu \hbox {mol/l}$) for 15 min. (**B**) The total concentration of free $\hbox {Ca}^{2+}$ was determined with a commercially available kit (Abcam, ab102505). Data are expressed as mean ± SEM. n = 5–6, *${p}<0.05$ vs. CTL.

We have previously demonstrated that HSP70 is key to PE-induced vascular contraction^4^. In Fig. 1A, we confirmed that, upon inhibition of HSP70, aortic rings challenged with a single dose of PE ($10\,\mu \hbox {mol/l}$) display a reduction in the force generated. Since vascular responses rely on the intracellular concentration of free $\hbox {Ca}^{2+}$, in this study, we measured the levels of this cation in control (CTL)- and VER155008-treated aortic samples stimulated with this $\alpha$-1 adrenergic agonist. We found that, compared with CTL samples, rings incubated with VER155008 have a substantial decrease in the total concentration of free $\hbox {Ca}^{2+}$ (Fig. 1B), which indicates that the mechanism targeted by HSP70 to influence vascular contraction might involve $\hbox {Ca}^{2+}$ dynamics.

### Impact of HSP70 blockade in PE-induced phasic contraction: role of IP3r-mediated intracellular $\hbox {Ca}^{2+}$ release

**Figure 2:**
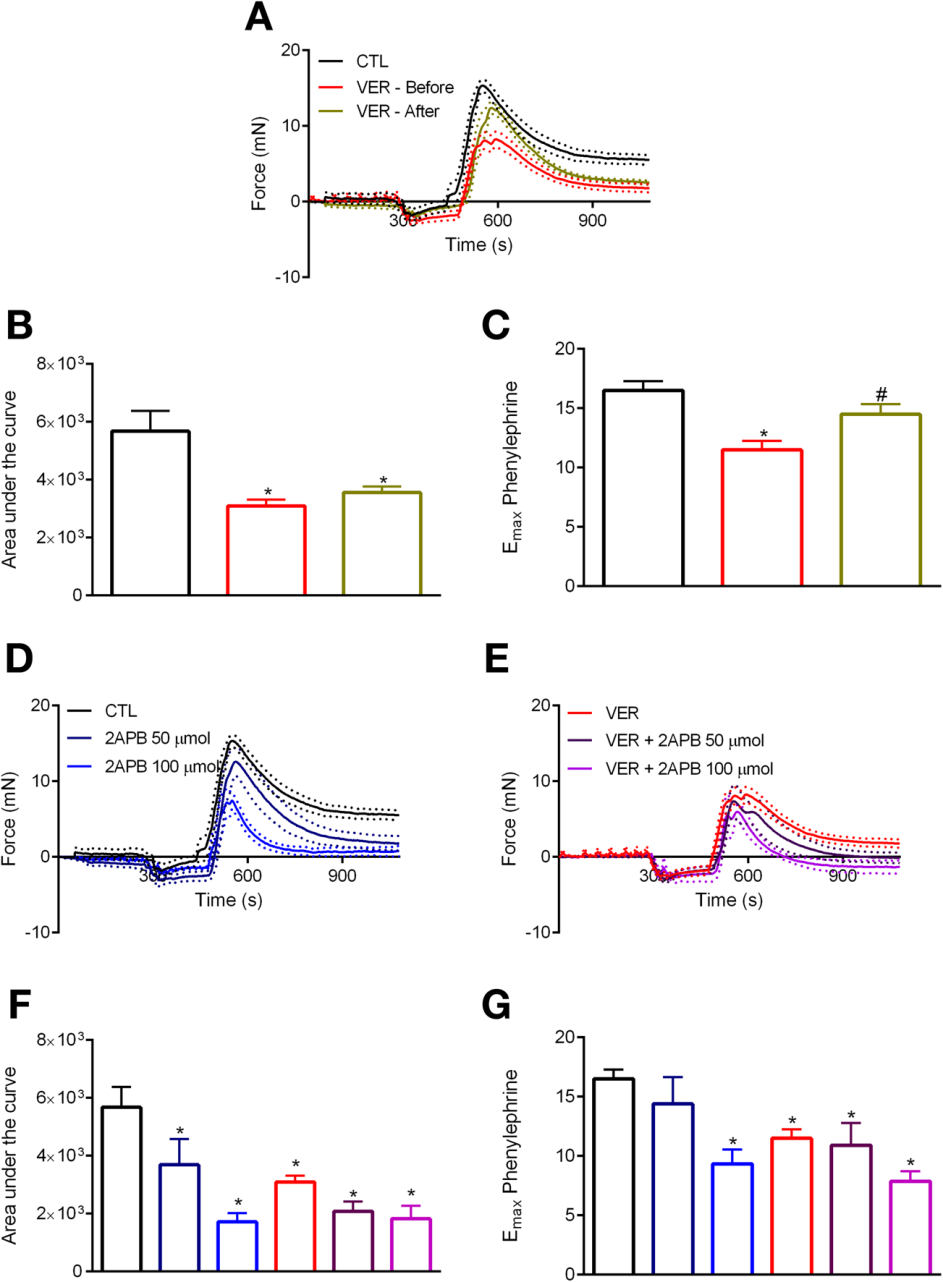
HSP70 contributes to phenylephrine-induced phasic contraction via IP3r-mediated intracellular $\hbox {Ca}^{2+}$ release. Aortic rings were challenged with phenylephrine ($10\,\upmu \hbox {mol/l}$) in 0[$\hbox {Ca}^{2+}$] Krebs’ solution in the presence of vehicle (CTL) or VER155008 ($10\,\upmu \hbox {mol/l}$, DMSO diluted), which was added before or after IP3r-mediated $\hbox {Ca}^{2+}$ release (**A**). In another set of experiments, samples were stimulated in the presence of (**D**) 2-aminoethoxydiphenyl borate (2-APB) (50 and $100\,\upmu \hbox {mol/l}$, DMSO diluted), or (**E**) the combination of 2-APB and VER155008. Inhibitors were added before IP3r-mediated $\hbox {Ca}^{2+}$ release. (**B**,**F**) Area under the curve and (**C**,**G**) $\hbox {E}_{\mathrm{max}}$. Panels (**B**,**C**) and (**F**,**G**) use the same color scheme as panels (**A**) and (**D**,**E**), respectively. Data are expressed as mean ± SEM. n = 14 for CTL and VER—before, n = 9 for VER—after, and n = 6 for all other groups, *${p}<0.05$ vs. CTL and #${p}<0.05$ vs. VER—before.

Inhibition of HSP70 decreases PE-induced phasic contraction in the aorta^4^. Here, to better understand this process, we performed functional studies in the absence of exogenous $\hbox {Ca}^{2+}$. Under this condition, PE induced a transient contractile response (Fig. 2A). Then, we confirmed that the blockade of HSP70 (VER—Before) decreases the total and the maximum response elicited by this agonist (Fig. 2B,C, respectively). Additionally, inhibition of maximum response only occurred if HSP70 was targeted before IP3r-mediated intracellular $\hbox {Ca}^{2+}$ release (Fig. 2C), which suggests that blockade of HSP70 attenuates phasic contraction by blunting the response elicited by this receptor.

Next, to build into this finding, we performed functional studies in the presence of 2-aminoethoxydiphenyl borate (2-APB), an inhibitor of the IP3r. Noteworthy, it is known that 2-APB also inhibits receptor- and store-operated $\hbox {Ca}^{2+}$ entry^23,24^, and therefore, this inhibitor can be used to study phasic and tonic vascular responses to $\alpha$-1 adrenergic stimulation^12^. In this set of experiments, aiming at blocking the IP3r, 2-APB was added at the moment we replaced the Krebs’ solution with 0[$\hbox {Ca}^{2+}$] Krebs’ solution. As expected, 2-APB produced a dose-dependent inhibitory effect (Fig. 2D). The hyporesponsive pattern detected in samples incubated with 2-APB was similar to that evoked by samples exposed to VER155008 (Fig. 2E vs. D). In subsequent experiments, rings were stimulated with PE in the presence of 2-APB and VER155008. The HSP70 inhibitor did not potentiate the total nor the maximum inhibitory effect of 2-APB (Fig. 2F,G, respectively), which, again, indicates that HSP70 affects PE-induced phasic contraction by acting upon IP3r-mediated mechanisms.

### Impact of HSP70 blockade in PE-induced tonic contraction: role of voltage-dependent and -independent $\hbox {Ca}^{2+}$ channels

**Figure 3:**
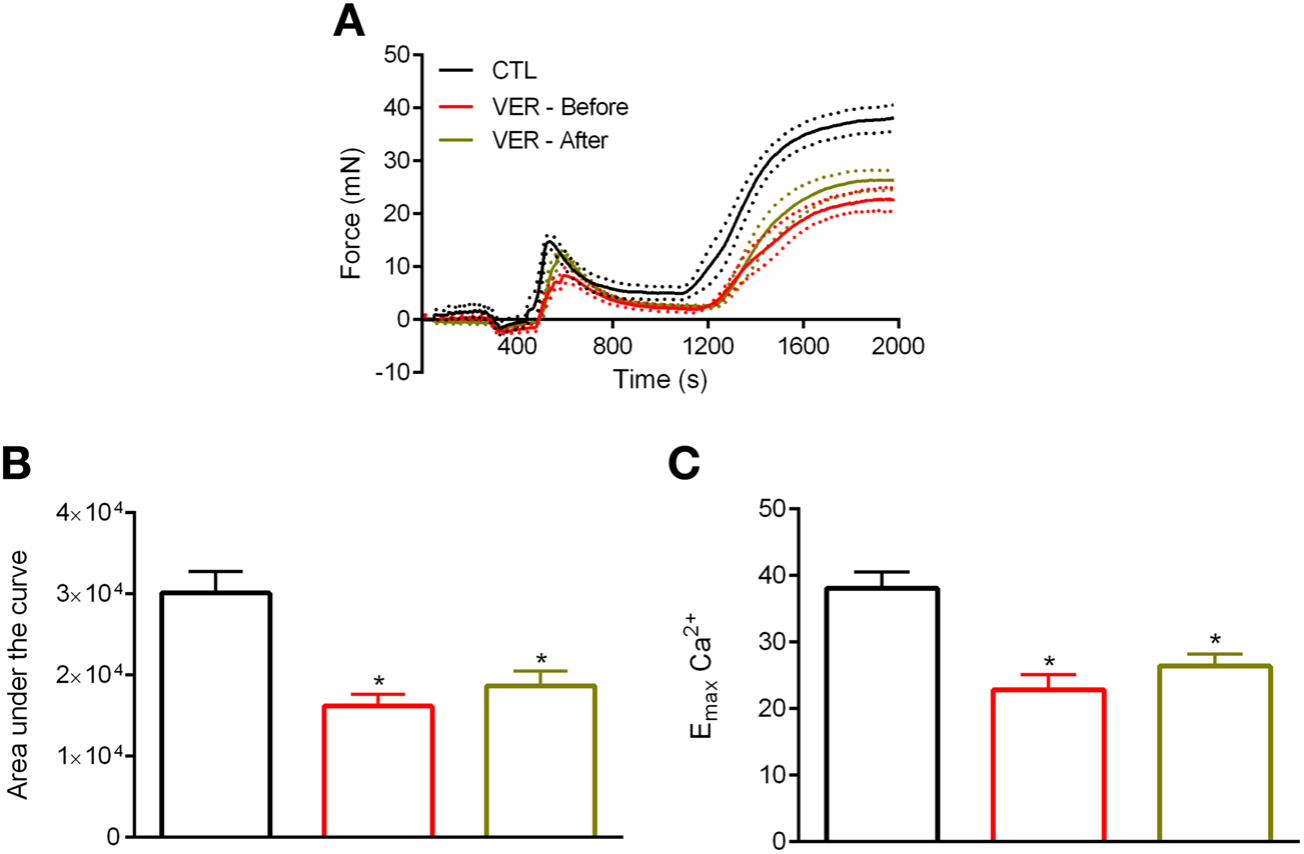
Inhibition of HSP70 attenuates tonic contraction in aorta stimulated with phenylephrine whether VER155008 is added before or after IP3r-mediated phasic contraction. (**A**) Aortic rings were challenged with phenylephrine ($10\,\upmu \hbox {mol/l}$) in 0[$\hbox {Ca}^{2+}$] Krebs’ solution in the presence of vehicle (CTL) or VER155008 ($10\,\upmu \hbox {mol/l}$, DMSO diluted), which was added before or after IP3r-mediated $\hbox {Ca}^{2+}$ release. The concentration of $\hbox {Ca}^{2+}$ was restored after 10 min, and the force developed was evaluated for 15 min. (**B**) Area under the curve and (**C**) $\hbox {E}_{\mathrm{max}}$. Panels (**B**,**C**) use the same color scheme as panel (**A**). Data are expressed as mean ± SEM. n = 9, *${p}<0.05$ vs. CTL.

Subsequently, we sought to identify the mechanism by which inhibition of HSP70 reduces PE-induced vascular tonic contraction. To accomplish this goal, we evaluated the force developed by aortic rings after restoring the initial concentration of $\hbox {Ca}^{2+}$ to the Krebs’ solution. The re-addition of $\hbox {Ca}^{2+}$ triggered a prolonged contraction phenotype in aortic rings (Fig. 3A). Then, in a first set of experiments, we added VER155008 before and after PE-induced IP3r-mediated contraction. The presence of VER155008 significantly decreased the total and the maximum response elicited by PE (Fig. 3B,C, respectively), and this hyporesponsive pattern was independent of the moment VER155008 was added to the chamber (i.e., whether it was before or after IP3r-mediated phasic contraction).

**Figure 4:**
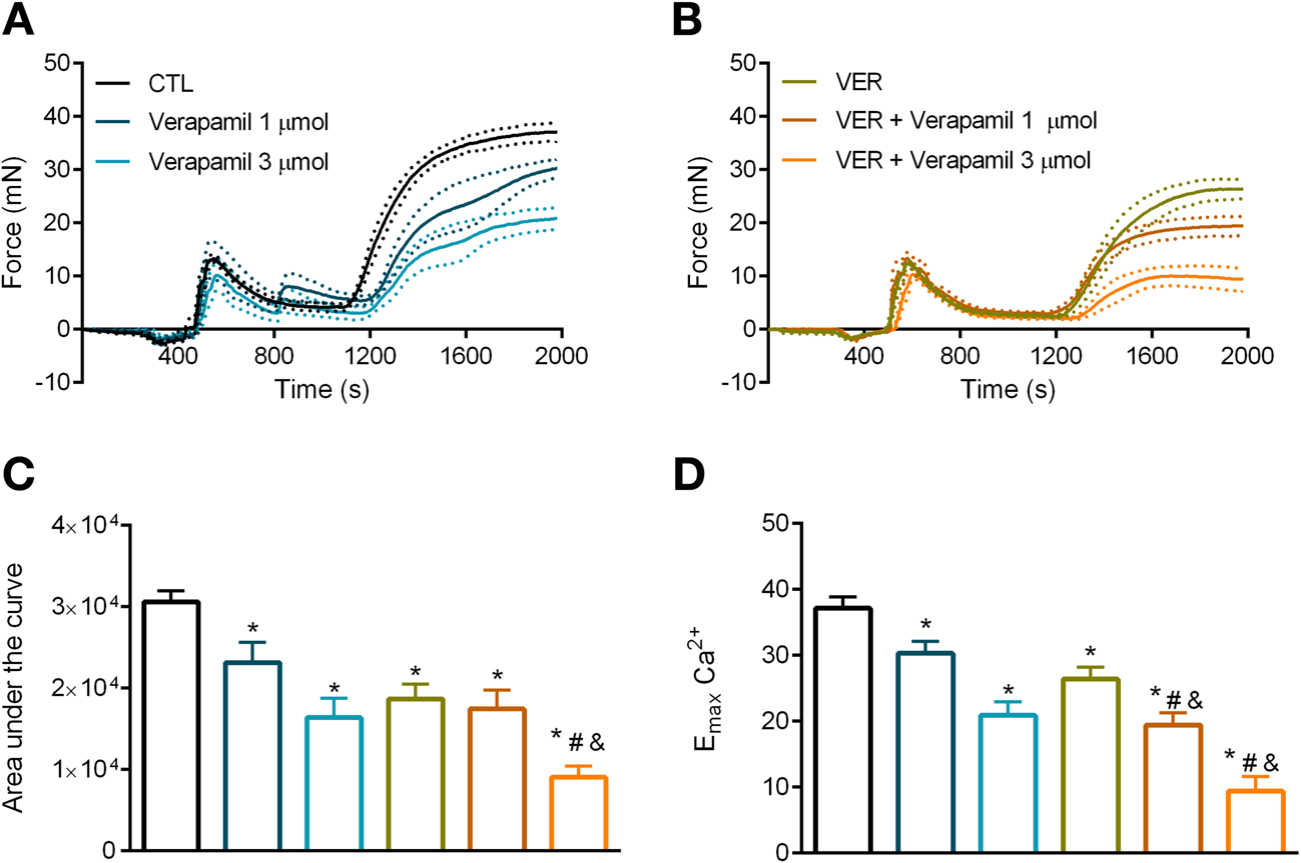
Blockade of HSP70 enhances the inhibitory effect of an L-type $\hbox {Ca}^{2+}$ channel blocker in aorta stimulated with phenylephrine. Aortic rings were challenged with phenylephrine ($10\,\upmu \hbox {mol/l}$) in 0[$\hbox {Ca}^{2+}$] Krebs’ solution in the presence of vehicle (CTL) or (**A**) verapamil (1 and 3 $\upmu$mol/l, DMSO diluted) or (**B**) VER155008 ($10\,\upmu \hbox {mol/l}$, DMSO diluted) as well as the combination of verapamil and VER155008. Inhibitors were added after IP3r-mediated $\hbox {Ca}^{2+}$ release. The concentration of $\hbox {Ca}^{2+}$ was restored after 10 min, and the force developed was evaluated for 15 min. (**C**) Area under the curve and (**D**) $\hbox {E}_{\mathrm{max}}$. Panels (**C**,**D**) use the same color scheme as panels (**A**,**B**). Data are expressed as mean ± SEM. n = 9 for CTL and VER and n = 5 for all other groups, *${p}<0.05$ vs. CTL, #${p}<0.05$ vs. verapamil, and&${p}<0.05$ vs. VER.

**Figure 5:**
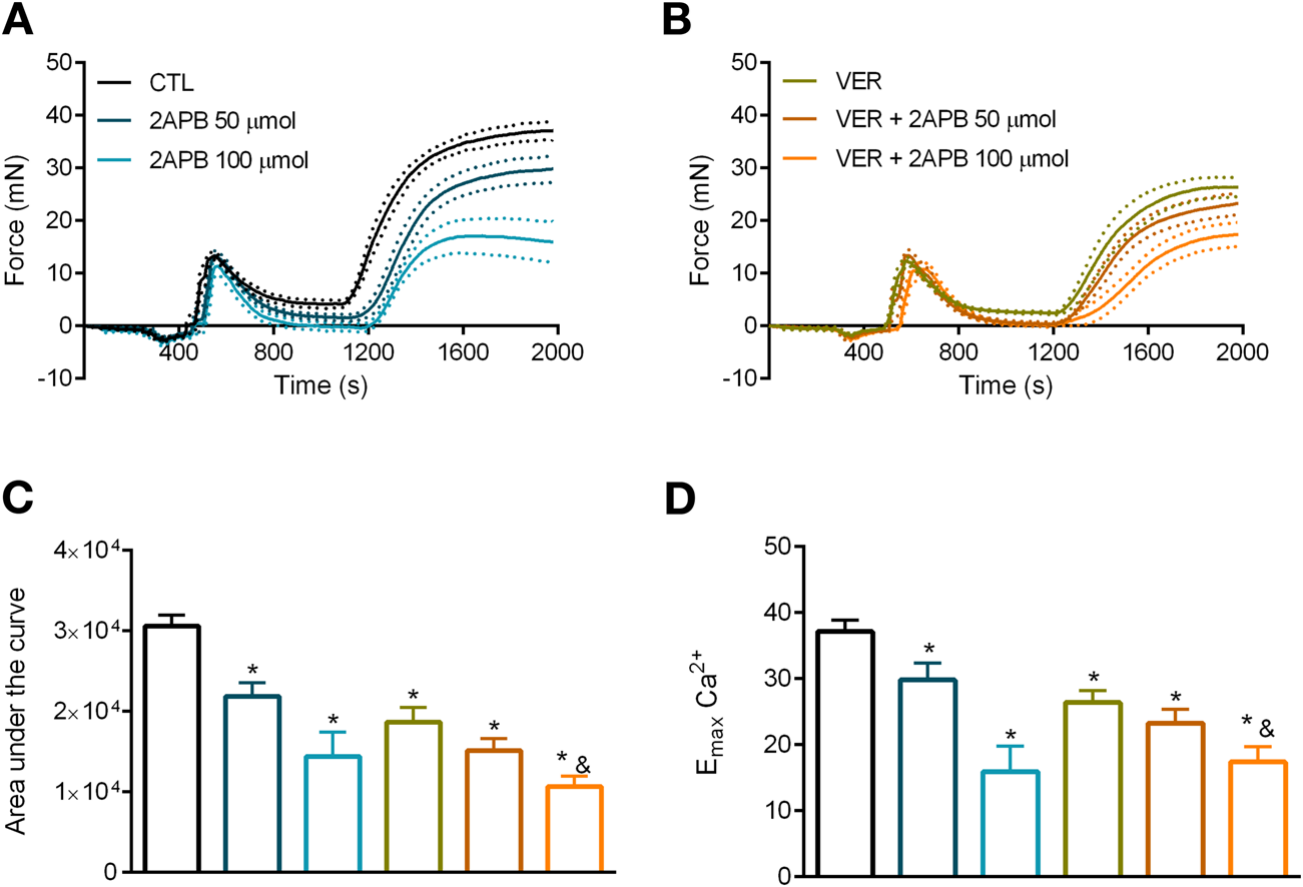
VER155008, an HSP70 inhibitor, does not augment the effect of a non-selective inhibitor of voltage-independent $\hbox {Ca}^{2+}$ channels in phenylephrine-stimulated aorta. Aortic rings were challenged with phenylephrine ($10\,\upmu \hbox {mol/l}$) in 0[$\hbox {Ca}^{2+}$] Krebs’ solution in the presence of vehicle (CTL) or (**A**) 2-aminoethoxydiphenyl borate (2-APB) (50 and 100 $\mu$mol/l, DMSO diluted) or (**B**) VER155008 ($10\,\upmu \hbox {mol/l}$, DMSO diluted) as well as the combination of 2-APB and VER155008. Inhibitors were added after IP3r-mediated $\hbox {Ca}^{2+}$ release. The concentration of $\hbox {Ca}^{2+}$ was restored after 10 min, and the force developed was evaluated for 15 min. (**C**) Area under the curve and (**D**) $\hbox {E}_{\mathrm{max}}$. Panels (**C**,**D**) use the same color scheme as panels (**A**,**B**). Data are expressed as mean ± SEM. n = 9 for CTL and VER and n = 6 for all other groups, *${p}<0.05$ vs. CTL and&${p}<0.05$ vs. VER.

Since $\hbox {Ca}^{2+}$ influx following $\alpha$-1 receptor stimulation is mediated by voltage-dependent and independent channels, we next investigated whether blockade of HSP70 impairs tonic contraction by interfering with these channels. Here, we show that the response exhibited by aortic rings, in the presence of VER155008 and following the re-addition of $\hbox {Ca}^{2+}$, was similar to the one observed in samples incubated with verapamil (Fig. 4B vs. A) or 2-APB (Fig. 5B vs. A). In fact, no statistical difference was observed between the use of the HSP70 inhibitor and verapamil or 2-APB in the total nor the maximum response elicited by the agonist (Figs. 4C,D; 5C,D, respectively). More interestingly, we observed that the combination of VER155008 and verapamil increased verapamil’s inhibitory effect, which reveals that the HSP70 inhibitor potentiates the effects of this L-type $\hbox {Ca}^{2+}$ channel (LTCC) blocker and points to these drugs impacting different mechanisms. Corroborating this statement, the combination of VER155008 and 2-APB, did not produce a synergism (Fig. 5B), which corroborates the notion that these drugs affect the same mechanism.

### Impact of HSP70 blockade in PE-induced phasic/tonic contraction: role of Rho-kinase and SERCA pump

**Figure 6:**
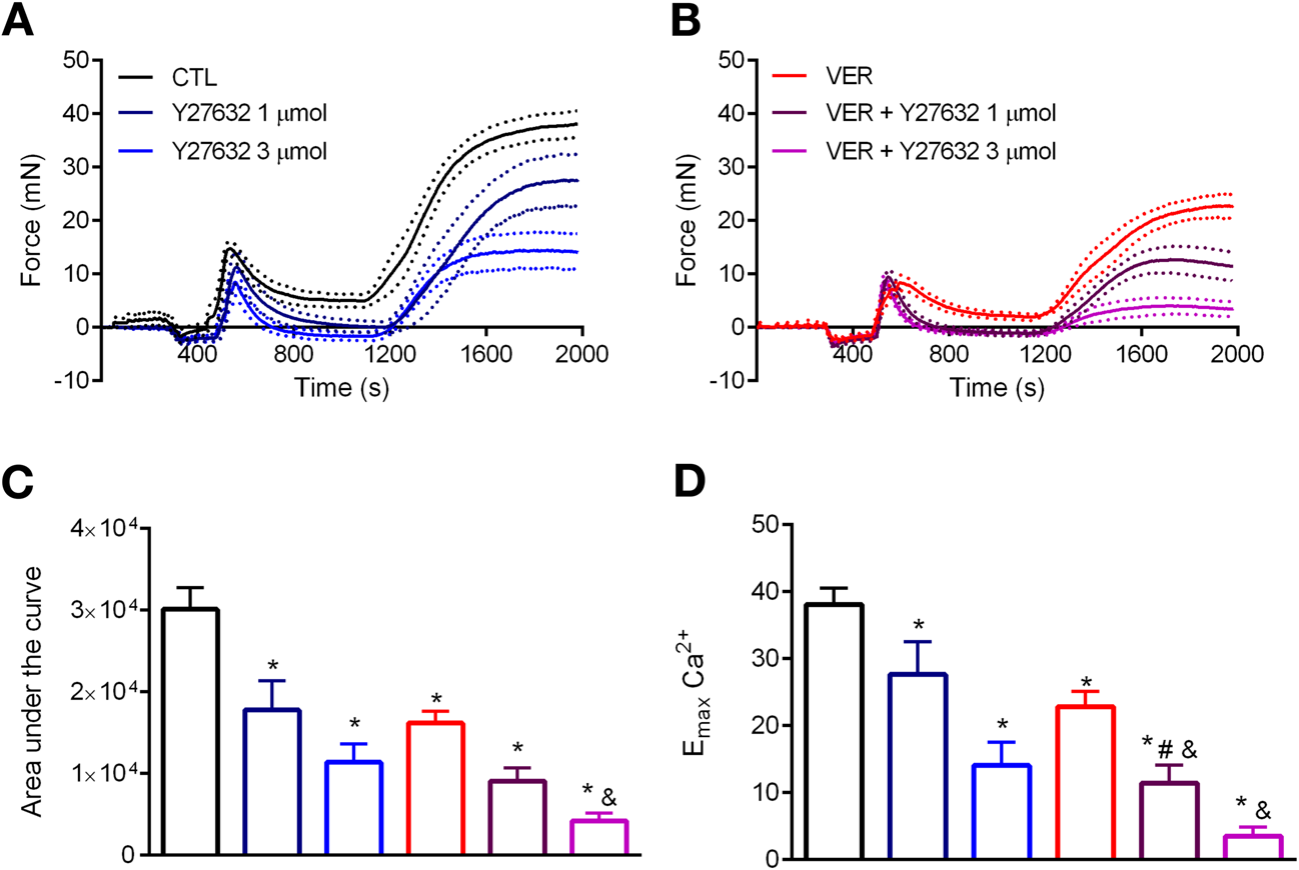
Blockade of HSP70 intensifies the inhibitory effect of a Rho-kinase inhibitor in aorta stimulated with phenylephrine. Aortic rings were challenged with phenylephrine ($10\,\upmu \hbox {mol/l}$) in 0[$\hbox {Ca}^{2+}$] Krebs’ solution in the presence of vehicle (CTL) or (**A**) Y27632 (1 and 3 $\mu$mol/l, DMSO diluted) or (**B**) VER155008 ($10\,\upmu \hbox {mol/l}$, DMSO diluted) as well as the combination of Y27632 and VER155008. Inhibitors were added before IP3r-mediated $\hbox {Ca}^{2+}$ release. The concentration of $\hbox {Ca}^{2+}$ was restored after 10 min, and the force developed was evaluated for 15 min. (**C**) Area under the curve and (**D**) $\hbox {E}_{\mathrm{max}}$. Panels (**C**,**D**) use the same color scheme as panels (**A**,**B**). Data are expressed as mean ± SEM. n = 9 for CTL and VER and n = 6 for all other groups, *${p}<0.05$ vs. CTL, #${p}<0.05$ vs. Y27632, and&${p}<0.05$ vs. VER.

Rho-kinase contributes to PE-induced contraction by promoting $\hbox {Ca}^{2+}$ sensitization^19–21^. Therefore, we next investigated the impact of a Rho-kinase inhibitor in the phasic and tonic contractions elicited by this agonist. The presence of Y27632, a Rho-kinase inhibitor, did not affect the phasic $\hbox {E}_{\mathrm{max}}$ of PE in 0[$\hbox {Ca}^{2+}$] Kreb’s solution (Fig. 6A). However, it consistently reduced the total response evoked by the drug (Fig. 6A). Similarly, the combination of Y27632 and VER155008 did not exacerbate the the drugs’ effect on maximum response, but it decreased the total response elicited by PE (Fig. 6B). Next, we concentrated our efforts to evaluate the effects of Y27632 and VER155008 in samples following the re-addition of $\hbox {Ca}^{2+}$ to the Kreb’s solution. The combination of Y27632 and VER155008 significantly reduced the drugs’ response in comparison with effects of these inhibitors independently (Fig. 6B vs. A), which suggests that these drugs are acting upon different mechanisms. Details about the total and maximum response elicited by phenylephrine can be found in Fig. 6C,D, respectively.

**Figure 7:**
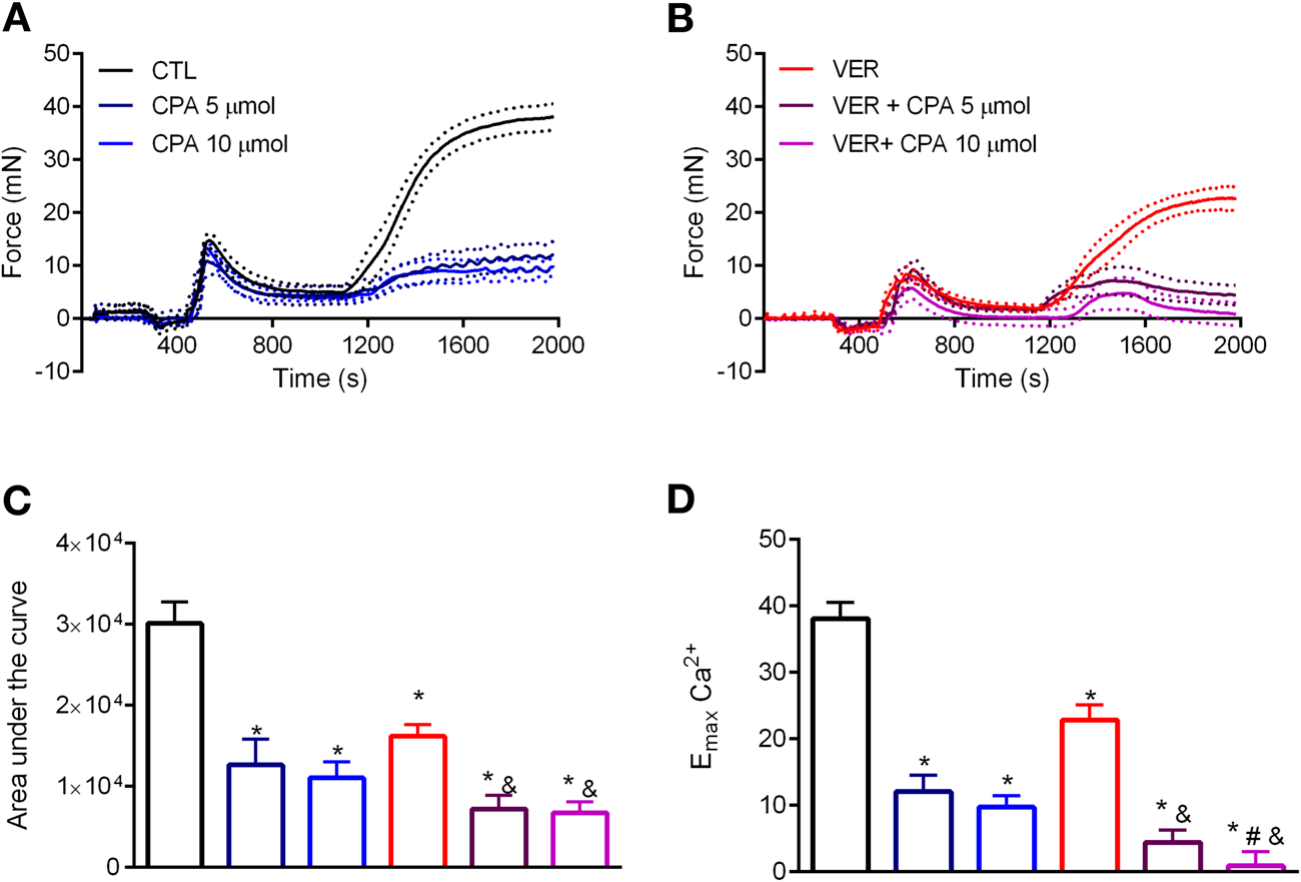
VER155008, an HSP70 inhibitor, further increases the effects of a SERCA pump inhibitor in aorta stimulated with phenylephrine. Aortic rings were challenged with phenylephrine ($10\,\upmu \hbox {mol/l}$) in 0[$\hbox {Ca}^{2+}$] Krebs’ solution in the presence of vehicle (CTL) or (**A**) cyclopiazonic acid (CPA) (5 and $10\,\upmu \hbox {mol/l}$, DMSO diluted) or (**B**) VER155008 ($10\,\upmu \hbox {mol/l}$, DMSO diluted) as well as the combination of CPA and VER155008. Inhibitors were added before IP3r-mediated $\hbox {Ca}^{2+}$ release. The concentration of $\hbox {Ca}^{2+}$ was restored after 10 min, and the force developed was evaluated for 15 min. (**C**) Area under the curve and (**D**) $\hbox {E}_{\mathrm{max}}$. Panels (**C**,**D**) use the same color scheme as panels (**A**,**B**). Data are expressed as mean±SEM. n = 9 for CTL and VER and n = 6 for all other groups, *${p}<0.05$ vs. CTL, #${p}<0.05$ vs. CPA, and&${p}<0.05$ vs. VER.

Up to this point, our results corroborate the notion that blockade of HSP70 weakens vascular contraction by decreasing the intracellular concentration of free $\hbox {Ca}^{2+}$. Thus, we next investigated if a SERCA pump inhibitor, which prevents $\hbox {Ca}^{2+}$ transfer from the cytoplasm to the SR, would affect the outcome measured in the presence of the HSP70 inhibitor. From our observations, it is clear that blocking the SERCA pump did not affect PE-induced phasic contraction, but it completely disrupted the vessels’ ability to develop tonic contraction (Fig. 7A). The latter was maximized in the presence of VER155008 (Fig. 7B), and points toward a complete impairment of $\hbox {Ca}^{2+}$ handling mechanisms in aortic rings. The results of the total and maximum response elicited by the agonist are presented in Fig. 7C,D, respectively.

## Discussion

In the present study, we expanded the concept that HSP70 is a role player in vascular physiology, as we show, for the first time, that proper vascular $\hbox {Ca}^{2+}$ handling stimulated by PE requires this protein. Our findings are of utmost importance because $\hbox {Ca}^{2+}$ is the chief mediator of vascular contraction and fluctuations in its intracellular levels modulate the contractile phenotype of blood vessels^1^. Therefore, notwithstanding the limitations of our study, it fills in a knowledge gap in vascular biology and it will, in turn, guide future research in this field, particularly due to the emergent link between HSP70 and cardiovascular/renal diseases^25–29^.

Throughout this study, we applied a well-established, yet indirect, protocol to investigate $\hbox {Ca}^{2+}$ changes in the presence of VER155008, which functions as an ATP-competitive inhibitor^30^. To overcome this limitation and strengthen our claims, a biochemical assay kit was also used to evaluate the free levels of this cation. In Fig. 1B, we specifically show that inhibition of HSP70 decreases the total levels of free $\hbox {Ca}^{2+}$, which ultimately, reduces the force of contraction. Here, it is important to recognize some pitfalls of this method. In recent years, researchers have evaluated $\hbox {Ca}^{2+}$ fluctuations with a fluorescent indicator, such as fura-2, and since the concentration of $\hbox {Ca}^{2+}$ rapidly change in vascular structures, it is considered a more accurate measurement for this cation. Additionally, in order to perform the biochemical assay, samples need to be homogenized and it might include mitochondrial $\hbox {Ca}^{2+}$, which does not affect vascular smooth muscle contraction. Still, we argue that the claim that blockade of HSP70 impairs vascular contraction by affecting $\hbox {Ca}^{2+}$ handling mechanisms are based on our collective findings, which include not only the indirect measurement of the free levels of $\hbox {Ca}^{2+}$, but also an extensive and well-detailed set of functional studies as well as evidence from previous studies. The literature shows a complex interaction between HSP70 and $\hbox {Ca}^{2+}$. In fact, the ATPase domain of HSP70 binds two $\hbox {Ca}^{2+}$ ions^9^ and changes in the intracellular concentration of this cation modulates the expression of HSP70^10,11^. Additionally, the genetic deletion of this protein impairs $\hbox {Ca}^{2+}$ homeostasis in cardiac and skeletal muscle^7,8^. Thus, our data elegantly builds upon previous knowledge as it uncovers a new biological process where HSP70 interacts with $\hbox {Ca}^{2+}$.

It is known that phasic contraction in response to PE in the aorta involves IP3r-mediated $\hbox {Ca}^{2+}$ release from the SR^12,15^. We previously demonstrated that, in vessels stimulated with this $\alpha -1$ agonist when HSP70 is blocked, there is a reduction in the amplitude of the fast component^4^. However, it was yet-to-be-determined if a direct relationship exists with the IP3r. Here, we confirmed that blockade of HSP70 also weakens PE-induced phasic contraction in aorta under 0[$\hbox {Ca}^{2+}$] Krebs’ solution (Fig. 2A). Similar results were also detected in samples incubated with 2-APB, an inhibitor of the IP3r (Fig. 2D). Interestingly, we found that the combination of VER155008 and 2-APB does not augment the latter’s inhibitory effect (Fig. 2E vs. D). Such findings indicate both inhibitors acting upon similar mechanisms. Corroborating this statement, we also detected that, if we block HSP70 after PE-mediated IP3r-induced phasic $\hbox {E}_{\mathrm{max}}$, the impact of VER155008 is abolished (Fig. 2A,C), which strongly suggests that HSP70 contributes to PE-induced phasic contraction via IP3r-mediated $\hbox {Ca}^{2+}$ release. In a counterintuitive manner, it has been previously demonstrated that upregulation of HSP70 reduces IP3r protein levels following ischemia/reoxygenation in PC12 cells^31^. Here, it is important to consider that $\hbox {Ca}^{2+}$ overload can occur during ischemia/reoxygenation^32^, and as discussed by the authors, the ultimate outcome observed was that HSP70 contributes to maintaining $\hbox {Ca}^{2+}$ homeostasis in these cells^31^. In this sense, our results align with the previous literature as we also show that the precise control of $\hbox {Ca}^{2+}$ handling requires HSP70.

Next, we turned our attention to try at understanding the mechanism(s) by which HSP70 affects vascular tonic contraction. A previous study demonstrated that PE-induced tonic contraction includes $\hbox {Ca}^{2+}$ influx via voltage-dependent and independent channels^12^. While there is limited information about an interaction between HSP70 and LTCC, it has been suggested that HSP70 might act by inhibiting voltage-gated $\hbox {Ca}^{2+}$ channel to prevent $\hbox {Ca}^{2+}$ overload, and consequently, apoptosis^33^. However, as highlighted by the authors experimental evidence was lacking. Here, we found that the HSP70 inhibitor potentiates the inhibitory effect of an LTCC blocker (Fig. 4B vs. A), but not of a non-selective inhibitor of voltage-independent $\hbox {Ca}^{2+}$ channels (Fig. 5B vs. A). Therefore, our data corroborate the idea that HSP70 contributes to tonic contraction by acting upon voltage-independent $\hbox {Ca}^{2+}$ channel-facilitated $\hbox {Ca}^{2+}$ influx. In support of this statement, we also confirmed that VER155008 reduces tonic contraction independently of the moment it is added to the chamber (i.e., whether it was before or after IP3r-mediated phasic contraction) (Fig. 2). Noteworthy, we used 2-APB to target voltage-independent $\hbox {Ca}^{2+}$ channels, which has an inhibitory effect towards NSCC and CRAC channels^12,34^. Consequently, we are unable to pinpoint the exact channel targeted by the HSP70 inhibitor. Another possibility one should consider in this context is the fact that a previous study has demonstrated that the constitutive HSP70 interacts with lipid membranes leading to the generation of a functional ATP-dependent cationic pathway^35^. Therefore, further studies are required to uncover the precise mechanism by which HSP70 targets $\hbox {Ca}^{2+}$ influx in PE-stimulated aorta.

Subsequently, we focused on determining the contribution of Rho-kinase, which promotes $\hbox {Ca}^{2+}$ sensitization, and therefore, affects the contractile phenotype of vascular structures^19,20^. From our data, it is clear that the combination of VER155008 with Y27632 amplifies the hyporesponsive pattern observed in the aorta in comparison with blocking these proteins independently (Fig. 6). Given our findings regarding the role of HSP70 in $\hbox {Ca}^{2+}$ influx, one can argue that these results were to be expected, especially because Rho-kinase impacts vascular contraction by inhibiting the myosin light chain phosphatase, which, in turn, prevents relaxation^36,37^. Therefore, it appears that two different mechanisms were targeted in this set of experiments. Corroborating this statement, a previous study found that heat shock-mediated vascular hypercontractility does not directly involve Rho-kinase^38^.

Finally, we investigated a potential interaction between HSP70 and the SERCA pump, which mediates $\hbox {Ca}^{2+}$ re-uptake by the SR^22^. In this study, we used the SERCA pump inhibitor, cyclopiazonic acid (CPA), which does not affect phasic contraction^15^. In fact, it has been demonstrated that it increases the intracellular concentration of $\hbox {Ca}^{2+}$ without changing the force of contraction^12^. Interestingly, in the presence of CPA, there is a shift in the contractile phenotype of vascular smooth muscle to rely mainly on $\hbox {Ca}^{2+}$ influx via voltage-independent $\hbox {Ca}^{2+}$ channels^12^. Here, as showed by others, we confirmed that CPA does not impact PE-induced phasic contraction (Fig. 7). More interestingly, we found that, under the conditions examined in this study, it disrupts the ability of the vessel to elicit tonic contraction following re-addition of $\hbox {Ca}^{2+}$ (Fig. 7). It is important to consider the importance of $\hbox {Ca}^{2+}$ re-uptake by the SR, as the influx of this ion is gated by its depletion from the SR^16^. When we combined the HSP70 inhibitor with CPA, we observed a complete impairment of the vessels’ ability to elicit contraction, which could be due to (a) the effects of CPA upon $\hbox {Ca}^{2+}$ re-uptake and/or (b) the effects of VER155008 on voltage-independent $\hbox {Ca}^{2+}$ channels, which was, under this condition, the main source of $\hbox {Ca}^{2+}$ influx. While, to the best of our knowledge, data regarding an interplay between HSP70 and the SERCA pump in vascular structures are nonexistent, the literature shows that this chaperone has a protective role towards this protein^33^. For example, in cardiomyocytes, the deletion of HSP70 associates with a decrease in the expression of SERCA2a^7^ whereas, in PC12 cells, overexpression of HSP70 increases the levels of SERCA2a and SERCA2b^31^. Importantly, it has been demonstrated that, in HEK-293 cells, HSP70 prevents thermal inactivation of SERCA2a, potentially by decreasing its oxidation and nitrosylation^39^. Likewise, HSP70 prevents the thermal inactivation of SERCA1a in fast-twitch skeletal muscle^40^. Nevertheless, these studies differ in many aspects from our work, especially the fact that we aimed at investigating this interaction in the absence of a pathological condition.

**Figure 8:**
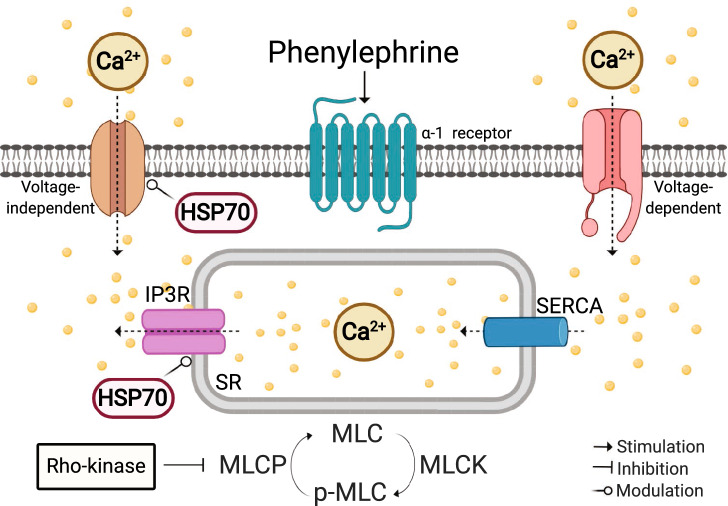
Proposed mechanism of HSP70 in aorta stimulated with phenylephrine. Pharmacological blockade of HSP70 modulates $\hbox {Ca}^{2+}$ handling mechanisms in aorta stimulated with phenylephrine potentially via crosstalk with (**a**) IP3r-mediated $\hbox {Ca}^{2+}$ release from the SR (phasic) and (**b**) voltage-independent $\hbox {Ca}^{2+}$ channels-facilitated $\hbox {Ca}^{2+}$ influx (tonic). Created with BioRender.com.

In summary, we showed that blockade of HSP70 affects $\hbox {Ca}^{2+}$ handling mechanisms in aorta stimulated with PE via crosstalk with (a) IP3r-mediated $\hbox {Ca}^{2+}$ release from the SR (phasic) and (b) voltage-independent $\hbox {Ca}^{2+}$ channels-facilitated $\hbox {Ca}^{2+}$ influx (tonic) (Fig. 8). There are, however, many points to enlighten, including the molecular aspects guiding this process. Here, we took an indirect approach to evaluate the role of HSP70 in vascular contraction, and therefore, further studies employing in situ enhancement of HSP70 in vascular smooth muscle are still required, since they could shed much light on the exact mechanisms involved in HSP70-mediated arterial contraction. A striking question arising at this point is whether the interaction between HSP70 and $\hbox {Ca}^{2+}$ remains in vascular diseases, such as diabetes and hypertension. Nevertheless, such a development in our understanding shifts the way one might approach disease-associated vascular complications, especially because we provided evidence that, under the conditions evaluated in this study, HSP70 contributes to vascular $\hbox {Ca}^{2+}$ dynamics, and $\hbox {Ca}^{2+}$ is a key player in healthy and diseased states.

## Methods

### Ethics statement

All animal procedures followed the Guide for the Care and Use of Laboratory Animals from the National Institutes of Health and were reviewed and approved by the Institutional Animal Care and Use Committees of Augusta University and Florida Institute of Technology.

### Animals

Sprague Dawley male rats were acquired from Charles River Laboratory and Taconic Biosciences. Animals were housed at room temperature with light exposure cycles of 12 h and free access to food and water. Rats were sacrificed with 10–12 weeks under isoflurane anesthesia (5% in 100% $\hbox {O}_{2}$). We carefully excised the aorta of each animal, and placed it in ice-cold Krebs’ solution (mmol/l: 130 NaCl, 4.7 KCl, 1.18 $\hbox {KH}_{2}\hbox {PO}_{4}$, 1.18 $\hbox {MgSO}_{4}$ * $7\hbox {H}_{2}\hbox {O}$, 14.9 $\hbox {NaHCO}_{3}$, 5.6 Dextrose, 1.56 $\hbox {CaCl}_{2}$ * $\hbox {H}_{2}\hbox {O}$, 0.026 EDTA). Then, the vessel was cleansed of fat tissue and cut into rings of 2 mm in length. Rings were immediately used in the $\hbox {Ca}^{2+}$ assay or in functional studies.

### Drugs and solutions

All pharmacological inhibitors and chemicals used in this study were purchased from Sigma-Aldrich (St. Louis, MO, United States). Stock solutions were diluted in DMSO and kept at − 20$\,^{\circ }$C.

### $\hbox {Ca}^{2+}$ assay

Aortic rings were incubated with vehicle or VER155008 ($10\,\upmu \hbox {mol/l}$) for 30 min in an isolated muscle bath covered in Krebs’ solution and gassed with carbogen (95% $\hbox {O}_{2}$ and 5% $\hbox {CO}_{2}$). Samples were then stimulated with PE ($10\,\upmu \hbox {mol/l}$) for 15 min, and quickly frozen in liquid nitrogen. Then, using the manufacturer’s instructions, we determined the intracellular concentration of free $\hbox {Ca}^{2+}$ with a commercially available kit (Abcam, ab102505).

### Functional studies

The isometric contraction of aortic rings was determined in a wire myograph (Danish Myograph Technology, Aarhus, Denmark). Briefly, aortic rings were mounted with a preload tension of 15 mN/mm in an isolated chamber containing 5 ml of Krebs’ solution ($37\,^{\circ }$C) aerated with carbogen (95% $\hbox {O}_{2}$ and 5% $\hbox {CO}_{2}$). To ensure viability, after a stabilization period of 1 h , we challenged the rings with a high KCl solution (120 mmol/l) until a “steady state” curve was generated (approximately 15 min). Then, rings were washed and allowed to equilibrate to the baseline tension. Subsequently, aortic samples were stimulated with a single dose of PE ($10\,\upmu \hbox {mol/l}$) for 15 min following incubation with vehicle of VER155008 ($10\,\upmu \hbox {mol/l}$) for 30 min.

In another set of experiments, the Krebs’ solution was replaced with $\hbox {Ca}^{2+}$ free (0[$\hbox {Ca}^{2+}$]) Krebs’ solution supplemented with 1 mmol/l EGTA. The contribution of specific mechanisms was assessed as follows:

- IP3r—vehicle or 2-APB (50 and $100\,\upmu \hbox {mol/l}$; IP3r inhibitor) or VER155008 ($10\,\upmu \hbox {mol/l}$) or the combination of 2-APB and VER155008 was added at time zero, which was the moment that the 0[$\hbox {Ca}^{2+}$] Krebs’ solution was placed into the chamber. After 3 min, samples were challenged with PE ($10\,\upmu \hbox {mol/l}$). Then, the time-force curves generated were evaluated for 10 min.
- Voltage-dependent and -independent $\hbox {Ca}^{2+}$ channels—0[$\hbox {Ca}^{2+}$] Krebs’ solution was placed into the myograph chamber. After 3 min, samples were challenged with PE ($10\,\upmu \hbox {mol/l}$). In this set of experiments, vehicle or VER155008 ($10\,\upmu \hbox {mol/l}$) or verapamil (1 and $3\,\upmu \hbox {mol/l}$; a selective LTCC blocker) or the combination of verapamil and VER155008 or 2-APB (50 and $100\,\upmu \hbox {mol/l}$; a non-selective inhibitor of voltage-independent $\hbox {Ca}^{2+}$ permeable channels) or the combination of 2-APB and VER155008 was added after the addition of PE. We added the inhibitors at this point because (a) VER155008 affects the amplitude of the phasic contraction elicited by PE^4^ and (b) 2-APB also affects the response of IP3r^23^. The time-force curves generated were evaluated for 10 min. Then, the extracellular $\hbox {Ca}^{2+}$ concentration was restored, and the force generated was evaluated for 15 min.
- Rho-kinase and SERCA pump—vehicle or Y27632 (1 and $3\, \upmu \hbox {mol/l}$; Rho-kinase inhibitor) or VER155008 ($10\,\upmu \hbox {mol/l}$) or the combination of Y27632 and VER155008 or CPA (5 and $10\,\upmu \hbox {mol/l}$; SERCA pump inhibitor) or the combination of CPA and VER155008 was added at time zero, which was the moment that the 0[$\hbox {Ca}^{2+}$] Krebs’ solution was placed into the chamber. Following 3 min, samples were challenged with PE ($10\,\upmu \hbox {mol/l}$). Then, the time-force curves generated were evaluated for 10 min. Subsequently, the extracellular $\hbox {Ca}^{2+}$ concentration was restored, and the tissue response was recorded for 15 min.

### Data and statistical analysis

The relationship between time (T) and force (F) expressed in the curve plots was calculated in the following way. We considered the moment we added 0[$\hbox {Ca}^{2+}$] Krebs’ solution into the chamber as $\hbox {t}_{0}$. At any point in time after $\hbox {t}_{0}$, F was calculated by subtracting the basal force, which is the observed measurement F at $\hbox {t}_{0}$. Then, we computed the area under the curve (AUC) and the $\hbox {E}_{\mathrm{max}}$ of the agonist. Results are expressed as mean ± SEM. Student t-test and one-way ANOVA followed by the Newman–Keuls method was used to determine statistical differences between two and three or more groups, respectively. n represents the number of animals and ${p}<0.05$ was considered statistically significant using the software GraphPad Prism, version 5.0.

## Data Availability

All animal data generated or analyzed during this study are included in this published article.
